# A Rare Case of Coexisting Medullary and Papillary Thyroid Carcinomas With Parathyroid Adenoma in Pancreatic Neuroendocrine Tumour (pNET)

**DOI:** 10.7759/cureus.99654

**Published:** 2025-12-19

**Authors:** Nazia Binte Salam, Fahmida Tithi, Sunil Zachariah

**Affiliations:** 1 General Internal Medicine, East Surrey Hospital, Redhill, GBR; 2 Diabetes and Endocrinology, East Surrey Hospital, Redhill, GBR

**Keywords:** endocrine, medullary thyroid carcinoma, multiple endocrine neoplasia, pancreatic neuroendocrine tumors, papillary carcinoma thyroid, thyroid cancer

## Abstract

We present a rare and complex case involving the simultaneous occurrence of medullary thyroid carcinoma (MTC), papillary thyroid carcinoma (PTC), and a parathyroid adenoma in a 79-year-old male. Additionally, incidental radiological findings suggest a pancreatic neuroendocrine tumour (NET). The coexistence of these diverse endocrine disorders is exceptionally uncommon and raises important questions regarding potential syndromic associations. Such cases highlight the necessity for thorough diagnostic evaluation, individualised treatment planning, and the collaboration of a multidisciplinary team, including endocrinologists, oncologists, surgeons, radiologists, and pathologists, to ensure optimal patient outcomes.

## Introduction

Pancreatic neuroendocrine tumours (pNETs) are uncommon malignancies, representing only 1-2% of all pancreatic neoplasms. Marini et al. found 10% of pNETs occur in the context of hereditary endocrine syndromes, notably multiple endocrine neoplasia type 1 (MEN1) [1]. The estimated annual incidence is one to two cases per 100,000 individuals. Kamei et al. mentioned that most pNETs are well-differentiated neoplasms that tend to grow slowly [2]. Li et al. classified pNETs into functional tumours, which secrete hormones that induce characteristic clinical syndromes, and nonfunctional tumours, which often lack overt hormonal manifestations [3]. Kamei et al. reported that early symptoms are usually vague or absent, that diagnosis is frequently delayed, and that many patients present with advanced disease [2].

McDonnell et al. mentioned MEN1 includes pancreatic tumours, pituitary tumours, and primary hyperparathyroidism. MEN2 (also known as MEN2A) consists of primary hyperparathyroidism, pheochromocytoma, and medullary thyroid cancer [4]. Additionally, Marfanoid habitus, pheochromocytoma, medullary thyroid cancer, and neuroma are seen in MEN3 (also known as MEN2B). Notably, there is an overlap between certain conditions and the MEN association. McDonnell et al. emphasized that a high index of diagnostic suspicion is required because of variable presentations of these conditions, and suspected patients should be promptly referred to a specialized centre for early initiation of treatment and regular follow-up [4]. MEN2, MEN3 and caused by mutations in the rearranged during transfection (RET) proto-oncogene. McDonnell et al. also stated that there is an established genotype-phenotype association in MEN2 and MEN3, but not in MEN1 [4].

McDonnell et al. mentioned that treatment depends on the cause. Surgical resection is the definitive treatment for parathyroid, pancreatic, and thyroid tumours [4]. Recently, medullary thyroid carcinoma (MTC) has been successfully treated with tyrosine kinase inhibitors. Vandetanib, an oral inhibitor of RET, vascular endothelial growth factor receptor (VEGFR) and epidermal growth factor receptor signalling, has shown long-lasting disease control in patients with advanced hereditary MTC.

Remarkably, about 95% of thyroid malignancies are differentiated thyroid carcinomas (DTCs), most commonly papillary thyroid carcinoma (PTC), which arises from follicular cells and accounts for nearly 85% of cases. Fallahi et al. found MTC arises from parafollicular C cells and is a distinct entity, with roughly 75% of cases occurring sporadically and 25% in hereditary forms, often linked to MEN types 2A and 2B [5]. Fallahi et al. also mentioned that the concurrent occurrence of PTC and MTC is rare [5]. Even more uncommon is the simultaneous presentation of MTC, PTC, parathyroid adenoma, and a suspected pancreatic NET.

## Case presentation

**Figure 1:**
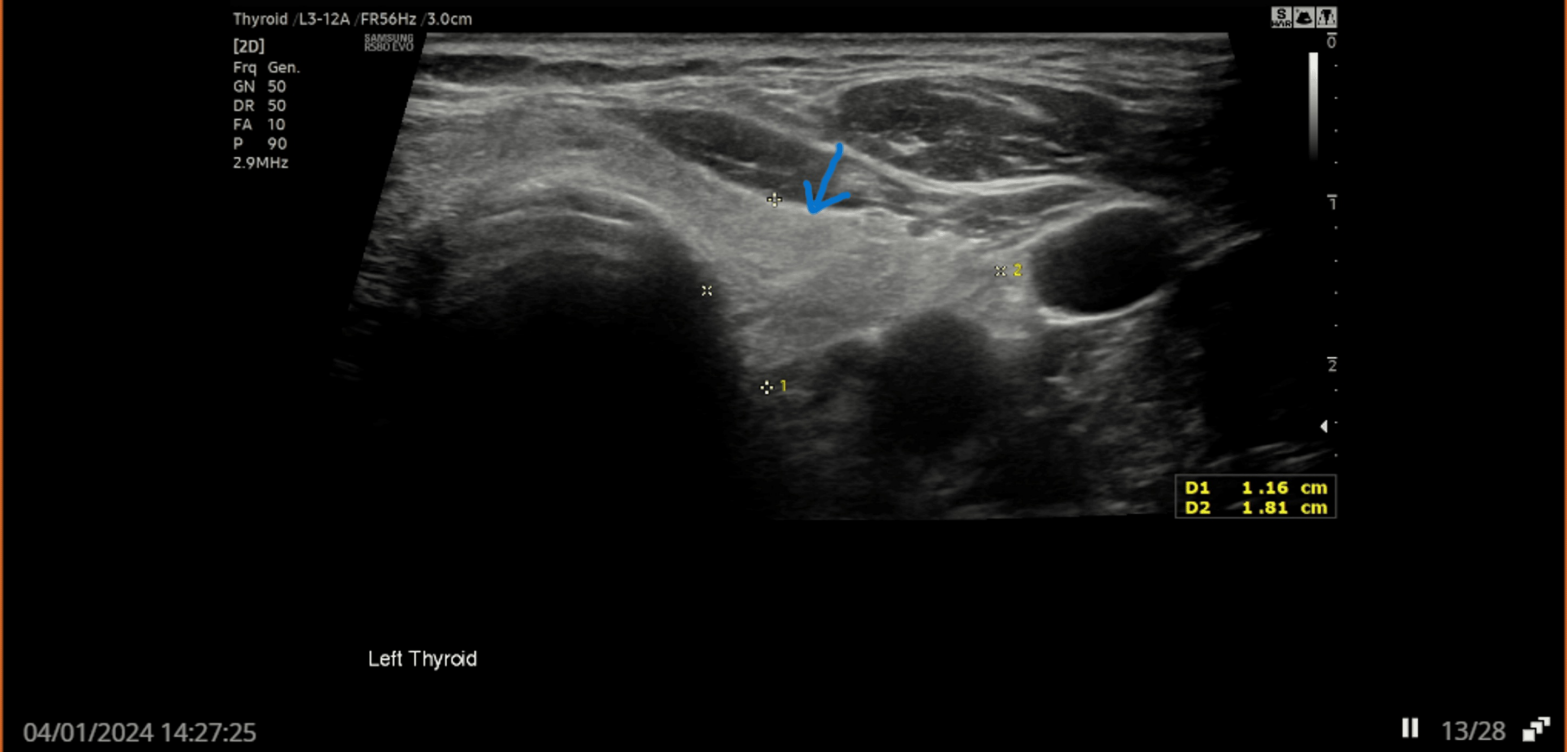
Ultrasound thyroid showing isoechoic U2 nodule in the left lobe Arrow showing isoechoic U2 nodule in left lobe of thyroid. U2: benign or non-cancerous nodule.

**Figure 2:**
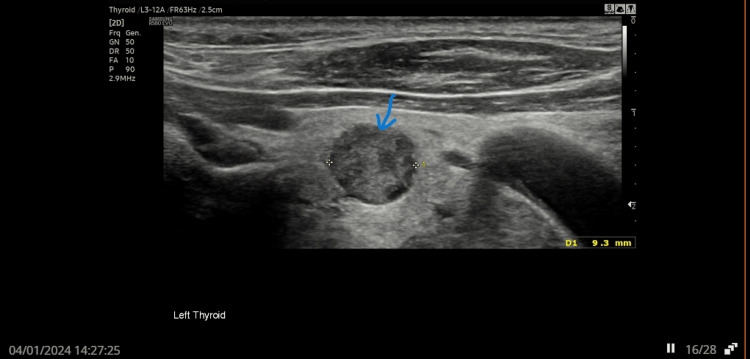
US thyroid showing hypoechoic U3 nodule in the left lobe Arrow showing hypoechoic U3 nodule in left lobe of thyroid. U3: equivocal nodule. The suspicion of malignancy is low to moderate.

**Figure 3:**
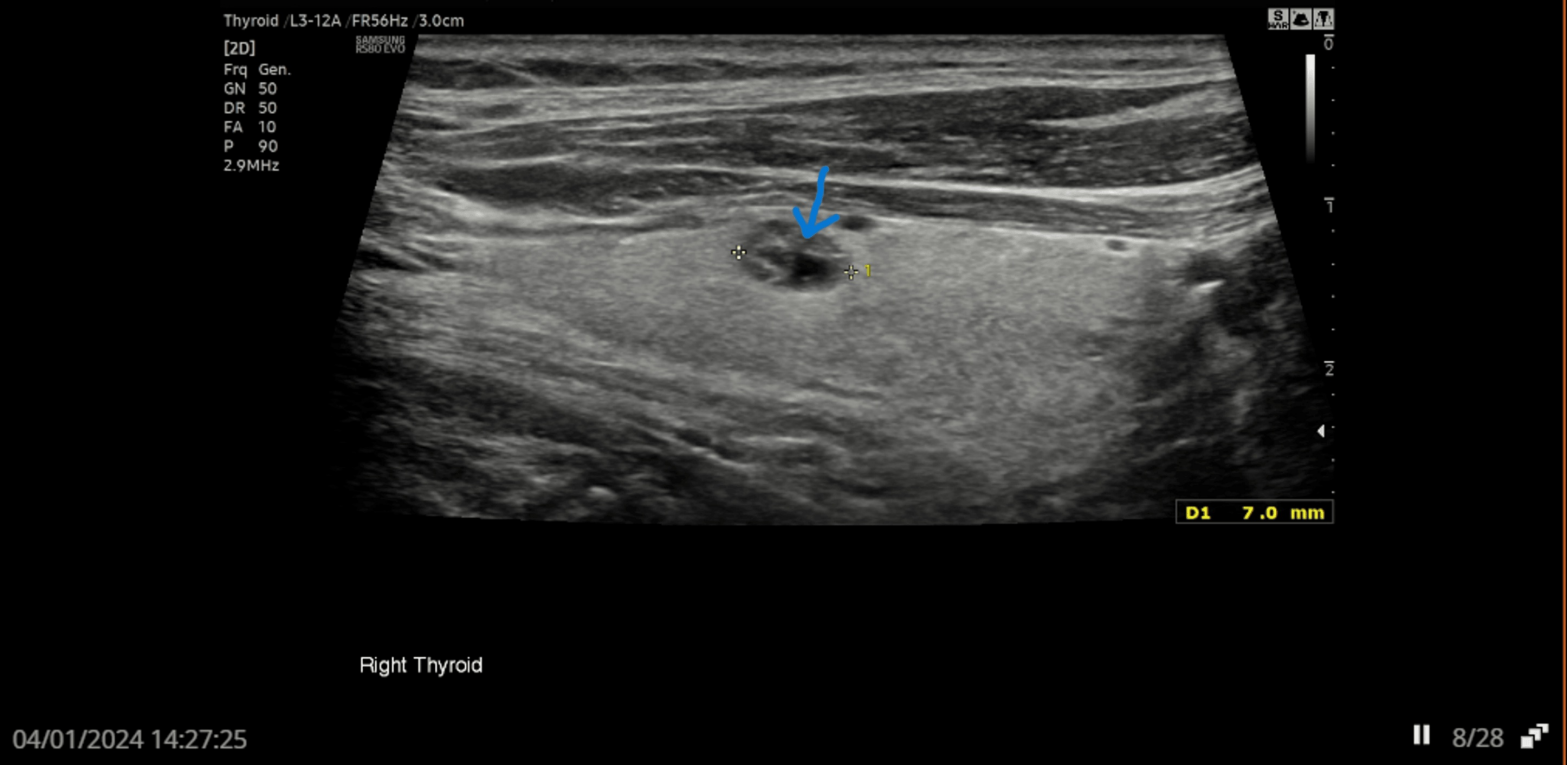
US thyroid showing hypoechoic nodule in right lobe Arrow showing hypoechoic nodule in right lobe of thyroid.

A 79-year-old man was referred to the endocrine team in early 2023 by his GP due to persistently elevated parathyroid hormone (PTH) levels, despite normal serum calcium and low phosphate. Initial thyroid ultrasound in June 2023 revealed an isoechoic U2 nodule in the left lobe (Figure 1) and hypoechoic U3 nodules in both lobes of the thyroid (Figures 2, 3). Follow-up imaging in January 2024 showed no significant change. 

**Figure 4:**
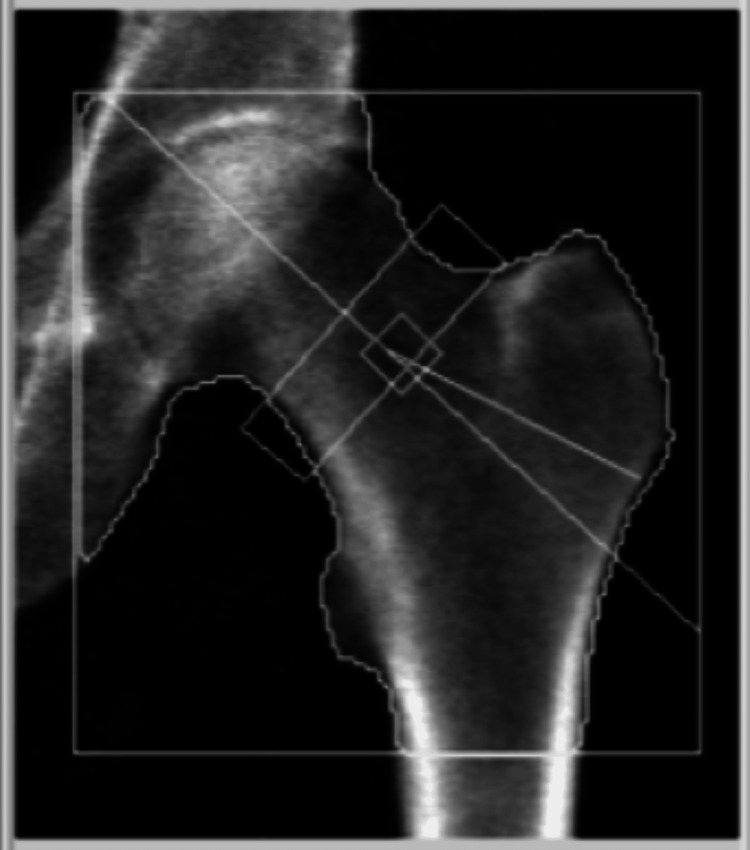
Dual X-ray absorptiometry (DEXA) scan image

He was asymptomatic, with no palpable neck mass or signs of compression, but was referred to Ear, Nose, and Throat (ENT) team via the two-week cancer pathway. Thyroid function tests, autoantibodies, calcium, vitamin D, and PTH levels were reassessed. Fine-needle aspiration cytology (FNAC) of the left U3 thyroid nodule in February 2024 showed features suspicious for medullary thyroid carcinoma or Hurthle cell lesion. Further blood tests revealed elevated calcitonin, carcinoembryonic antigen (CEA), persistently elevated PTH, and calcium (Table 1). Urinary metanephrines were negative. A dual X-ray absorptiometry (DEXA) scan in April 2024 revealed osteopenia (Figure 4, Tables 2, 3).

**Table 1:** Table showing the biochemistry CART: cocaine- and amphetamine-regulated transcript.

Biochemistry	Normal range	April 2023	September 2023	January 2024	March 2024	June 2024	July 2024
Parathyroid	1.6- 7.2 pmol/L	21.5	N/A	15.6	13.4	N/A	N/A
Serum Adjusted Calcium	2.2- 2.6 mmol/L	2.44	2.45	2.54	2.56	N/A	2.31
Serum Phosphate	0.8-1.5 mmol/L	0.65	0.75	0.64	0.80	N/A	N/A
Serum Carcinoembryonic antigen	0-4.9 ug/L	N/A	N/A	N/A	8.8	N/A	<1.7
Serum Calcitonin	0-11.8 ng/L	N/A	N/A	N/A	164	N/A	1.2
Vasoactive intestinal peptide	0-30 pmol/L	N/A	N/A	N/A	N/A	15	N/A
Pancreatic polypeptide	0-300 pmol/L	N/A	N/A	N/A	N/A	54	N/A
Gastrin	0-48 pmol/L	N/A	N/A	N/A	N/A	168	N/A
Glucagon	0-50 pmol/L	N/A	N/A	N/A	N/A	72	N/A
CART	0-129 pmol/L	N/A	N/A	N/A	N/A	38	N/A
Somatostatin	0-150 pmol/L	N/A	N/A	N/A	N/A	44	N/A
Chromogranin A	0-60 pmol/L	N/A	N/A	N/A	N/A	17	N/A
Chromogranin B	0-150 pmol/L	N/A	N/A	N/A	N/A	66	N/A
Urine non metadrenaline	0-3.3 umol/24h	N/A	N/A	N/A	2.02	N/A	N/A
Urine Metadrenaline	0-1.2 umol/24h	N/A	N/A	N/A	0.68	N/A	N/A
Urine 3 Methoxytyramine	0-2.5 0.68 umol/24h	N/A	N/A	N/A	0.68	N/A	N/A

**Table 2:** Result summary of dual X-ray absorptiometry (DEXA) scan Total Bone Mineral Density (BMD) Coefficient of Variation (CV) 1.0%, Area Correction Factor (ACF) = 1.023, Bone Correction Factor (BCF) = 0.988, Total Hip (TH) = 6.000. WHO Classification: Osteopenia [6]

Region	Area(cm^2^)	BMC[(g)]	BMD[g/cm^2^]	T-score	PR (Peak Reference)	Z-score	AM (Age Matched)
Neck	6.21	4.08	0.657	-2.0	71	-0.6	89
Total	45.04	39.39	0.875	-1.0	85	-0.1	98

**Table 3:** Table showing 10-year fracture risk estimated using WHO Fracture Risk Assessment Tool: FRAX WHO Fracture Risk Assessment Tool [7] Reported risk factors: UK, T-score (WHO): -1.7, BMI: 27.1, Secondary Osteoporosis

10-year Fracture Risk	
Major Osteoporotic Fracture	6.5%
Hip Fracture	2.5%

Tables 2, 3 showed osteopenia, prompting further assessment and an endocrine MDT discussion.

**Figure 5:**
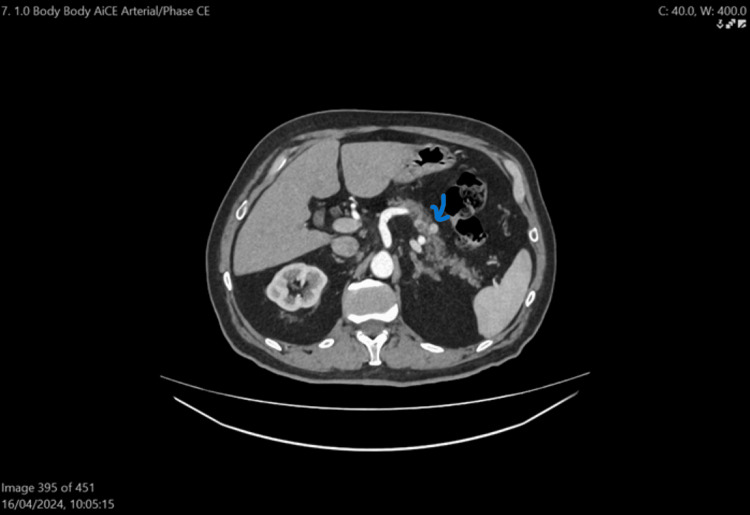
CT Neck Thorax Abdomen showing pancreatic nodule Arrow showing pancreatic nodule.

In 2024, a contrast-enhanced CT scan of the neck and thorax abdomen revealed no evidence of an aerodigestive mass. Previously known thyroid nodules were noted, along with mildly prominent lymph nodes at levels 3 and 4 on the left. A 9 mm suspected parathyroid lesion was identified along the left lateral wall of the oesophagus at the C7-T1 level. Additionally, a calcified nodule was observed in the right upper lobe of the lung, but no suspicious pulmonary lesions or lymphadenopathy were present. An 11 mm enhancing nodule in the pancreatic body (Figure 5) raised concern for malignancy, and a gastrointestinal review was recommended. Gallstones were also detected. There was no pleural effusion, pulmonary embolism, breast mass, or aggressive bone lesions.

The thyroid MDT in April 2024 recommended further assessment of the pancreatic lesion. An MRI pancreas showed an 8 mm lesion in the tail with a differential diagnosis of intrapancreatic accessory spleen versus NET. A PET dotatate scan in May 2024 confirmed two dotatate-avid pancreatic foci, indicative of well-differentiated NETs.

**Figure 6:**
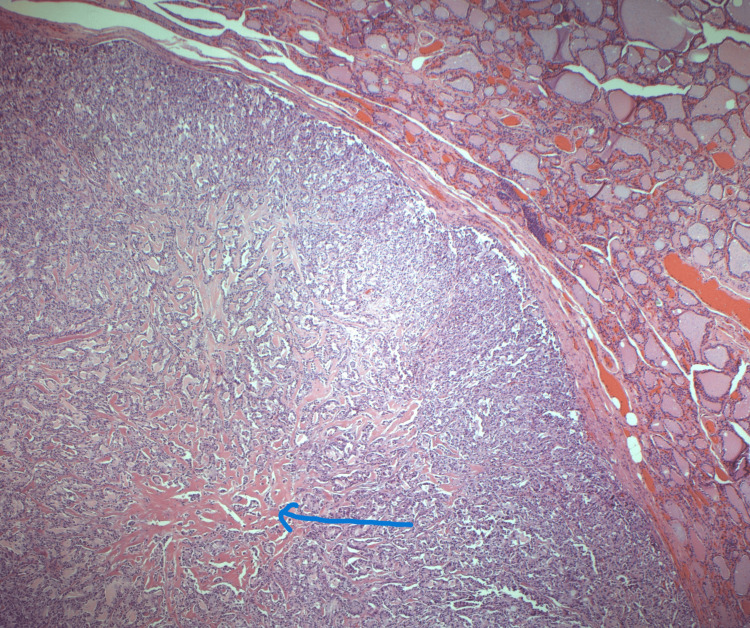
Histology image showing medullary thyroid carcinoma

The patient underwent total thyroidectomy with parathyroidectomy on 30th May 2024. Histological examination confirmed a 9 mm medullary thyroid carcinoma (Figure 6) in the left lobe (pT1aN0M0) with focal amyloid deposition. In addition, incidental bilateral papillary thyroid carcinomas (pT1aN0M0) were identified. A benign parathyroid adenoma was also noted. All 28 lymph nodes examined were negative for malignancy.

Postoperatively, the patient showed normalized biochemical markers: calcium, calcitonin, and CEA as of July 2024 (Table 1). Genetic testing in September 2024 was negative for RET mutations or MEN syndromes.

## Discussion

This case underscores a highly unusual confluence of endocrine pathologies. MTC accounts for only 3-4% of all thyroid cancers, and its coexistence with PTC - arising from entirely different cell lines - is rare, with few cases documented. Even rarer is the concurrent presence of a parathyroid adenoma and a suspected pancreatic NET, raising suspicion of MEN2, although genetic testing was negative.

The patient’s mildly elevated calcium, high PTH, and the CT finding of a lesion adjacent to the oesophagus were consistent with a parathyroid adenoma. Notably, his thyroid ultrasound, initially performed for parathyroid localisation, incidentally identified the U3 nodule that ultimately revealed MTC. This signifies the diagnostic value of incidental findings and broad-spectrum imaging.

High calcitonin level in this case favoured the diagnosis of MTC. Raised CEA also supported pancreatic and thyroid carcinoma. Parathyroid hormone level was three times the upper limit of normal, which came down after treatment. In summary, a combination of elevated calcitonin levels, Ultrasound findings, FNAC, and PET uptake supports the diagnosis of MTC and prompts treatment. PET dotatate avidity in the pancreas suggests the possibility of synchronous NETs. While histological confirmation was not yet available at the time of this report, the pancreatic lesions remain under surveillance by the upper gastrointestinal (GI) team.

Multidisciplinary collaboration between endocrinology, radiology, ENT, surgery, and upper GI specialists enabled timely and effective diagnosis and management. Importantly, the absence of genetic mutations indicates that this complex presentation may be sporadic rather than syndromic.

## Conclusions

This case illustrates the diagnostic challenges posed by multiple endocrine abnormalities. Incidental imaging of a thyroid nodule and biochemical screening revealed the syndromic-like presentations. This also reinforces that negative genetic testing does not exclude complex sporadic endocrine tumour combinations. Clinicians should be more aware of concurrent endocrine pathology when encountering biochemical-radiological discrepancies. The rare synchronous occurrence of MTC, PTC, a parathyroid adenoma, and a probable pancreatic NET underscores the importance of comprehensive evaluation and multidisciplinary collaboration. Despite the absence of a known hereditary syndrome, continued follow-up and surveillance are crucial given the potential for recurrence or additional endocrine pathology.
